# The leptin receptor gene affects piglet behavior and growth

**DOI:** 10.1093/jas/skad296

**Published:** 2023-09-02

**Authors:** Rafael Suárez-Mesa, Roger Ros-Freixedes, Marta Díaz, Júlia Marsellés, Ramona N Pena, Josep Reixach, Joan Estany

**Affiliations:** Department of Animal Science, University of Lleida – Agrotecnio-CERCA Center, 25198 Lleida, Catalonia, Spain; Department of Animal Science, University of Lleida – Agrotecnio-CERCA Center, 25198 Lleida, Catalonia, Spain; Selección Batallé S.A., 17421 Riudarenes, Catalonia, Spain; Selección Batallé S.A., 17421 Riudarenes, Catalonia, Spain; Department of Animal Science, University of Lleida – Agrotecnio-CERCA Center, 25198 Lleida, Catalonia, Spain; Selección Batallé S.A., 17421 Riudarenes, Catalonia, Spain; Department of Animal Science, University of Lleida – Agrotecnio-CERCA Center, 25198 Lleida, Catalonia, Spain

**Keywords:** leptin receptor, growth, lactation, maternal effects, pigs, vitality

## Abstract

Piglets with low birth weight present low vitality after farrowing, often leading to impaired weight gain during lactation. A recessive missense variant (C > T) for increased appetite and fatness in the porcine leptin receptor gene (rs709596309) causes a negative maternal effect on the weight of piglets at weaning. However, it is not known whether this variant already exerts an effect on the birth weight and vitality of newborn piglets and on their growing capacity during lactation. An experiment was conducted using 668 purebred Duroc piglets (131 CC, 311 CT, and 226 TT) from 74 multiparous sows (9 CC, 43 CT, and 22 TT) and 14 boars (1 CC, 10 CT, and 3 TT). All piglets were individually weighed at birth and tested for vitality, which was assessed on a scale from 1 (low vitality) to 3 (high vitality) based on behavioral observations, including the status of the piglet immediately before the test. Only non-adopted piglets were considered for piglet performance at weaning. Inferences on the effect of the genotype on birth and weaning traits were done on a Bayesian setting based on 2-trait bivariate models including the effects of the piglet and the litter, as well as the genotype of the sow and the piglet, the sex of the piglet, and the parity number. Vitality and the status of the piglet before the test were analyzed using a liability threshold (probit) model. As compared to other genotypes, TT newborn piglets were 28 g heavier, were more vital (the probability of being scored as highly vital was 6.5% higher) and were more often found suckling before the test (the probability of being suckling at test was 6.5% higher). As a result, they grew more during lactation (153 g) and were heavier at weaning (169 g) than littermates of the two other genotypes, thus partly compensating for the limited maternal capacity of TT sows. Our findings provide evidence that appetite-influencing genes, such as the leptin receptor gene, have developmental implications from very early life stages.

## Teaser

A variant in the leptin receptor gene influences the weight and vitality of piglets at birth and their growing potential until weaning.

## Introduction

Mothering and piglet’s abilities are limiting factors for improving performance in the nursing period. The probability of mortality and delayed growth in piglets is largely driven by low birth weight. Piglets with low birth weight present meager energy reserves and low vitality after farrowing, often leading to impaired weight gain during lactation and thus to low weight at weaning. This can be particularly challenging in litters from sows with limited maternal capacity, which usually are those that do not mobilize enough body reserves to support milk production needs.

Leptin is a hormone predominantly secreted by white adipocytes that regulates food intake and energy balance. Leptin deficiency causes excessive feed intake and energy savings and, consequently, greater body weight and fat mass. Defective leptin receptor expression produces similar obese phenotypes. In pigs, there is a recessive missense mutation in the leptin receptor (*LEPR*) gene (Óvilo et al., 2005) that increases overall fatness (Ros-Freixedes et al., 2016) and it is known to be at the origin of an antagonism between maternal and direct effects on body weight. Thus, Solé et al. (2021b) found that the homozygous recessive sows present a negative maternal effect on carcass weight of a similar magnitude to the positive effect that this genotype has on each individual pig. In an independent experiment, Solé et al. (2021a) confirmed the negative maternal effect of recessive homozygous sows, showing that they also deliver lighter litters at weaning. In line with Solé et al. (2021b), this result can be primarily attributed to the fact that recessive homozygous sows are less prone to mobilize body reserves into milk. We do not know, however, if the effects of this mutation are already apparent at birth, thus influencing the weight and vitality of newborn piglets and their growing capacity until weaning. Therefore, the aim of this study was to determine whether *LEPR* influences the weight of piglets from birth to weaning, as well as its relationship with piglet vitality.

## Material and Methods

Data were collected in a commercial herd following applicable regulations and good practice guidelines on the protection of animals kept for farming purposes. In compliance with European Directive 2010/63/EU and Spanish Royal Decree 53/2013, the present study did not imply any invasive procedure or treatment to the animals. The Ethics Committee for Animal Experimentation of the University of Lleida reviewed and approved the study (CEEA 04-06/21).

### Animals and Experimental Design

The experiment was conducted on a farm of purebred Duroc pigs from the same genetic line (Solanes et al., 2009) managed using standard practices. All animals used in the study were genotyped for the *LEPR* single nucleotide polymorphism (rs709596309; C > T) as described by Solé et al. (2021b). A total of 74 multiparous sows (9 CC, 43 CT, and 22 TT) on their first oestrus after weaning were inseminated with semen of 14 sires (1 CC, 10 CT, and 3 TT) to produce 767 live piglets. Sows and sires were mostly heterozygotes to maximize allele segregation. The sows were fed gestation (2181 kcal net energy, 12.8% crude protein, 5.9% crude fiber, 2.5% crude fat, and 0.7% total lysine per kg as fed) and lactation (2360 kcal net energy, 15.8% crude protein, 4.5% crude fiber, 4.0% crude fat, and 1.1% total lysine per kg as fed) cereal-based commercial diets (Esporc, Riudarenes, Girona, and Spain) according to the feeding program described by Piles et al (2020). All deliveries happened within a 6-wk interval from August 8, 2021. Within 24 h of birth (12 h, SD 7), available piglets were individually weighed (**BW**) and tested for vitality (see below). Subsequently, litter size was equalized by cross-fostering to 10 to 11 piglets per litter (10.6 piglets, SD 1.2), ensuring that litter features were homogeneous between TT and C − (CC and CT) sow genotypes (Table 1). Male piglets were castrated within the first week of age and creep feed was offered to all litters from 10 d after birth until weaning (20.2 d, SD 0.8), at which time piglets were weighed again to determine the weight at weaning (**WW**) and the weight gain during lactation (**WG**). For the purpose of this study, only the 668 piglets that could be genotyped for *LEPR* (131 CC, 311 CT, and 226 TT) were included in the analyses. Of these, only those that were raised with their biological mother (non-adopted) were considered for piglet performance at weaning (118 CC, 263 CT, and 206 TT). Backfat and loin thickness at 5 cm off the midline at the position of the last rib using a portable ultrasonic scanner (Piglog 105; Frontmatec, Kolding, Denmark) were measured in all the sows around the day of farrowing (−0.7 d, SD 2.9) and after mid-lactation (16.7 d, SD 1.6).

**Table 1.** Mean (SD) for litter features by sow *LEPR* genotype

Trait	*LEPR* genotype	
	TT	C−
No. of sows	22	52
No. of sires^1^	11	13
Farrowing number	3.9 (1.1)	3.9 (0.9)
No. of total piglets born	11.8 (2.6)	11.1 (2.8)
No. of piglets born alive	11.0 (2.4)	10.1 (2.6)
No. of suckling piglets	10.5 (1.0)	10.7 (1.3)
No. of weaned piglets	9.2 (1.1)	9.3 (1.5)
No. of non-adopted weaned piglets	8.4 (1.7)	7.9 (2.3)

^1^Of these, 10 sires were mated with sows of both genotypes.

### Assessment of Piglet Vitality

Vitality was assessed using a behavioral rating scale. According to the results by Muns et al. (2013), vitality (**VIT**) was measured on a scale from 0 to 3 as the sum of the score of the 2 most relevant behavioral traits for piglet body weight gain at weaning and survival (udder stimulation and number of completed circles around an enclosure). For the observations, piglets were individually separated from the litter and introduced to a 55-cm diam. × 60-cm height, solid, plastic enclosure, open at the bottom and top. Each piglet was given a score on (i) udder stimulation (1 or 0), depending on whether or not the piglet showed head movements, emulating udder stimulation activity or searching behavior, within 30 s, and on (ii) the number of completed circles around the enclosure during 30 s (0 to 2). A circle was considered completed if the piglet was able to turn its body axis 360° from its initial orientation or walk along the limits of the bucket once within 30 s. In total, the duration of the test was around 10 min per sow. Immediately before starting the test, the status of the piglet (**ST**) was noted, including if it was suckling, active (walking, standing, and others) or inactive (sitting and sleeping). Since only three piglets had a VIT of 0, they were merged into the VIT score 1 category. Thus, VIT categories were summarizes as low (1), medium (2), and high (3).

### Statistical analyses

The effect of the *LEPR* genotype (TT vs C–) on birth (BW, VIT, and ST) and weaning (WW and WG) traits was estimated fitting 2-trait bivariate models, where one of the traits was always BW. Correlations between any other two traits were estimated similarly with the corresponding 2-trait bivariate models. The traits associated with body weight (BW, WW, and WG), which were assumed normally distributed, were described by a linear model including the effects of the *LEPR* genotype of the sow (TT and C–) and the piglet (TT and C––, or the three genotypes), the sex of the piglet (male and female), and the parity number (2 to 6) as systematic effects, as well as the piglet and the litter. In contrast, categorical behavioral traits (VIT and ST) were analyzed using a liability threshold (probit) model (Sorensen and Gianola, 2002), which assumes an underlying normal distributed liability that, over a given threshold, produces a positive outcome. Here, liability was explained by the same linear model as above. The number of total piglets born (or weaned piglets) was included as a covariate in the model for BW, VIT, and ST (or for WW and WG). The effect of three technicians that performed the vitality test was also accounted for in the model for VIT and ST.

Inferences were done on Bayesian setting using TM software (Legarra et al., 2011; http://genoweb.toulouse.inra.fr/~alegarra/tm_folder [deposited: 3 August 2011]). Data augmentation was performed to analyze the data. Observed data, liability, and missing records imputed by data ­augmentation were assumed to be conditionally normally distributed as follows:


$$\begin{array}{l} \left[\begin{array}{l} y_{BW}\;y_{2}\; \end{array}\right]\;|b_{BW},b_{2},a_{BW},a_{2},c_{BW},c_{2},R∼\;N\;\left(X\left[\begin{array}{l} b_{BW}\;b_{2}\; \end{array}\right]+Z\left[\begin{array}{l} a_{BW}\;a_{2}\; \end{array}\right]+W\left[\begin{array}{l} c_{BW}\;c_{2}\; \end{array}\right],\;R\right), \end{array}$$

where **y**_i_ is the vector of augmented data for trait i (BW and others); **b**_i_, **a**_i_, and **c**_i_ are the vectors of systematic, additive genetic, and litter effects, respectively; ***X***_i_, ***Z***_i_, and ***W***_i_, the incidence matrices that relate **b**_i_, **a**_i_, and **c**_i_ with **y**_i_, respectively, and ***R*** the residual (co)variance matrix. Sorting records by trait, and pig within trait, ***R*** could be written as ***R***_0_ ⊗ ***I***, with ***R***_0_ being the 2 × 2 residual (co)variance matrix between BW and the other trait and ***I*** an identity matrix of appropriate order. Additive genetic and litter values, conditionally on the associated (co)variance components, were both assumed multivariate normally distributed with mean 0 and with (co)variance ***G*** ⊗ ***A*** and ***C*** ⊗ ***I***, respectively, where ***A*** was the numerator relationship matrix calculated with a pedigree of 668 piglets from 74 sows and 14 sires, and ***G*** and ***C*** were the 2 × 2 (co)variance matrices of genetic and litter effects between the 2 traits. Flat priors bounded at a very large value (*M*) were used for systematic effects (−*M*, *M*) and the additive genetic, litter and residual (co)variances (0, *M*), excepting the residual variance for binary outcomes, which was set to 1. Based on the normal assumptions, the sow’s backfat and loin thickness before and after parity was analyzed with a univariate linear model including the *LEPR* genotype of the sow and the parity number.

Marginal posterior distributions for all unknowns were estimated using Gibbs sampling (Gelfand and Smith, 1990). Statistical inferences (namely, posterior means and SD, and posterior probabilities of differences being greater or lower than 0 [P_0_]) were derived from the samples of the marginal posterior distribution using a unique chain of 1,000,000 iterations, where the first 200,000 were discarded and 1 sample out of 1,000 iterations was retained. Convergence was tested using the *Z*-criterion of Geweke and visual inspection of convergence plots. We considered that there was a strong (or moderate or suggestive) evidence of difference between effects when *P*_0_ was at least 0.95 (or 0.90 or 0.85, respectively).

## Results

### Effect of LEPR on piglet growth

In line with the effect of the recessive T allele, TT sows had 2.1 mm (P_0_ = 0.95) more backfat thickness at farrowing than C − sows (Table 2). However, this excess of backfat at farrowing did not alter the amount of fat lost during lactation. The *LEPR* polymorphism influenced the piglet’s growth pattern (Table 3). In comparison with C − littermates, TT piglets were heavier both at birth (+28 g, *P*_0_ = 0.85, for BW) and at weaning (+169 g, *P*_0_ = 0.93, for WW), although the relative impact of this difference was higher at weaning (3.7% of the mean, for WW, and 1.8% of the mean, for BW). This was due to a greater weight gain during lactation of TT piglets (+153 g, P_0_ = 0.94, for WG). No difference between CC and CT piglets was found for BW (+2.2 g, *P*_0_ = 0.51) or for WW (−4.9 g, *P*_0_ = 0.49). A different trend was observed for sex differences, with males being heavier than females at birth (+53 g, *P*_0_ = 0.99), but not at weaning (+49 g, *P*_0_ = 0.71). Weight gain during lactation did not differ between males and females.

**Table 2.** Mean of the trait and mean (SD) of the marginal posterior distribution of the difference between sow *LEPR* genotypes (TT vs C−) for backfat and loin thickness at birth and at weaning^1^

Trait^2^		Genotype	
	Mean	TT—C−	*P*_0_
Backfat thickness at parity, mm	25.0	+2.1 (1.2)	0.95
Loin thickness at parity, mm	41.8	−0.8 (1.4)	0.73
Backfat thickness at weaning, mm	22.9	+1.1 (1.2)	0.83
Loin thickness at weaning, mm	41.0	−0.5 (1.2)	0.68
Backfat thickness loss during lactation, mm	2.1	+0.2 (0.4)	0.69
Loin thickness loss during lactation, mm	0.9	−0.4 (1.3)	0.64

^1^ *P*_0_: posterior probability of the difference between genotypes or sexes being greater (if positive) or lower (if negative) than zero.

^2^Backfat and loin thickness at farrowing (weaning) was adjusted for litter weight at birth (weaning). Backfat and loin thickness loss during lactation was adjusted for litter weight gain during lactation.

**Table 3.** Mean of the trait and mean (SD) of the marginal posterior distribution of the difference between maternal (sow) and direct (piglet) *LEPR* genotypes (TT vs C−) and between sexes for birth and weaning weight

Trait	Mean	Genotype				Sex	
		Sow _TT- C−_	*P*_0_ ^1^	Piglet_TT-C−_	*P*_0_	Male–female ^2^	*P*_0_ ^1^
Birth weight, g	1,538	−3 (50)	0.53	+28 (26)	0.85	+53 (22)	0.99
Weaning weight, g	4,585	−155 (175)	0.82	+169 (115)	0.93	+49 (97)	0.71
Weight gain during lactation, g	3,064	−94 (152)	0.73	+153 (100)	0.94	0 (86)	0.53

^1^ *P*_0_: posterior probability of the difference between genotypes or sexes being greater (if positive) or lower (if negative) than zero.

^2^Males were castrated within the first week of age.

As in previous studies, TT sows provided an unfavorable maternal environment to piglets (Table 2), although, due to the limited number of sows involved here, this effect was not fully captured (−155 g, *P*_0_ = 0.82, for WW). For this reason, we reassessed the effect the *LEPR* polymorphism on piglet’s growth by considering only the piglets born to CT sows. Using this subset of data, results were confirmed and TT piglets continued to weigh more at weaning than C − piglets (+189 g, *P*_0_ = 0.90, for WW). The same happened when the analysis was performed including the adopted piglets (+145 g, *P*_0_ = 0.91).

### Effect of LEPR on piglet behavior

**Figure 1.**
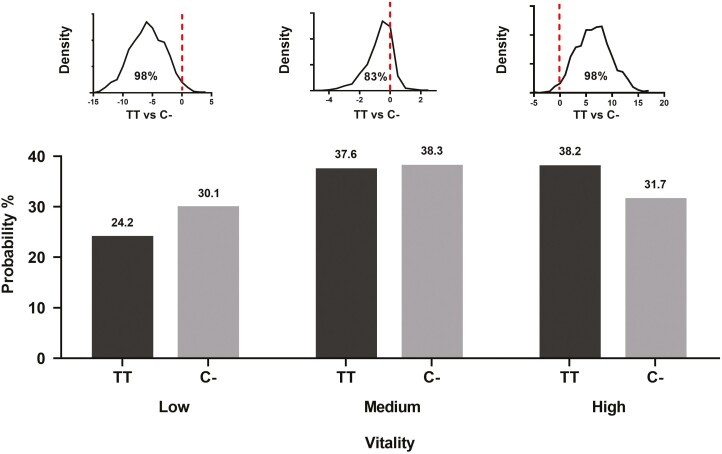
Probability of a piglet showing low, medium, and high vitality by *LEPR* genotype (TT and C−). Values represented are adjusted for systematic effects. The marginal posterior distribution of the difference between TT and C– genotypes is depicted on the top of each comparison, with the red-dotted line indicating the zero (no difference) and the accompanying percentage standing for the posterior probability of TT being higher or lower than C– (area under the curve at the right or left side of the line, respectively).

A differential behavior can be argued as another reason why TT piglets performed better during lactation. *LEPR* genotype and sex differences for VIT and ST on the liability scale are given in Table 4. In terms of VIT, TT piglets were more vital than C − piglets (+0.18, *P*_0_ = 0.98). As for growth traits, no difference was observed between CC and CT piglets for VIT −-0.04, *P*_0_ = 0.64). The increased vitality of TT piglets is made evident by a shift to higher VIT categories (Figure 1). Thus, given the average liability values, the probability that TT piglets score as highly vital is 6.5% higher than in C − piglets (38.2%, for TT, vs 31.7%, for C−). In contrast, TT piglets are 5.8% less likely to be scored as lowly vital than C − littermates (24.2% vs 30.1%). We also analyzed the status of the piglet immediately before the VIT test and we found that TT piglets were more likely found suckling (35.7% vs 29.1%, *P*_0_ = 0.89) or inactive (30.1% vs 24.1%, *P*_0_ = 0.91) than C − piglets. If not suckling, however, they were less active (i.e., wandering) than their C − littermates (27.2% vs 39.6%, *P*_0_ = 0.99). In contrast, these probabilities were reversed for TT sows, which had more active piglets (40.0% vs 26.9%, *P*_0_ = 0.93) than C − sows. Sexes did no differ for VIT, but females showed a higher probability to be suckling than males (36.3% vs 28.6%, *P*_0_ = 0.97).

**Table 4.** Mean of the trait and mean (SD) of the marginal posterior distribution of the difference between maternal (sow) and individual (piglet) *LEPR* genotypes (TT vs C−) on the liability scale for behavior traits at birth.

Trait^2^	Mean	Genotype				Sex	
		Sow_TT-C−_	*P*_0_ ^1^	Piglet_TT-C−_	*P*_0_	Male–female	*P*_0_ ^1^
Vitality score	0.61	−0.11 (0.15)	0.79	+0.18 (0.09)	0.98	+0.04 (0.08)	0.70
Suckling at test	−0.54	−0.34 (0.32)	0.86	+0.17 (0.14)	0.89	−0.22 (0.12)	0.97
Active at test	−0.44	+0.36 (0.24)	0.93	−0.34 (0.15)	0.99	+0.01 (0.12)	0.52
Inactive at test	−0.61	−0.02 (0.26)	0.53	+0.18 (0.14)	0.91	+0.21 (0.12)	0.96

^1^ *P*_0_: posterior probability of the difference between genotypes or sexes being greater (if positive) or lower (if negative) than zero.

^2^Values expressed in units of residual standard deviation.

Behavioral traits at birth showed evidence of genetic variability (Table 5), with heritabilities ranging from 0.13, for suckling piglets, to 0.30, for VIT, values that are within the range of those observed for BW and WW, respectively. The outcome of the vitality test was little influenced by the status of the piglet at test. The correlations of VIT with the liability values for inactive (−0.33, SD 0.05), active (0.10, SD 0.05), and suckling (0.20, SD 0.05) status were rather low. Behavioral traits at birth were also weakly associated with BW (Table 5). Only the correlation of BW with VIT (−0.17, *P*_0_ > 0.99) and active status at test (−0.10, *P*_0_ = 0.97) were different from zero. No relationship was found of VIT and status at birth with performance at weaning (WW and WG).

**Table 5.** Posterior means (SD) of heritability and phenotypic correlations for birth weight, weaning weight, and behavior traits at birth

Trait	Heritability	Phenotypic correlation		
		Birth weight	Weaning weight	Weight gain
Birth weight	0.17 (0.14)	—	0.50 (0.03)	0.27 (0.04)
Weaning weight	0.27 (0.11)	—	—	0.97 (0.01)
Weight gain during lactation	0.31 (0.11)	—	—	—
Vitality score	0.30 (0.13)	−0.17 (0.04)	−0.06 (0.05)	−0.03 (0.05)
Suckling at test	0.13 (0.07)	0.04 (0.05)	0.03 (0.05)	0.02 (0.05)
Active at test	0.26 (0.09)	−0.10 (0.05)	−0.05 (0.06)	−0.03 (0.06)
Inactive at test	0.14 (0.08)	0.06 (0.05)	0.04 (0.06)	0.03 (0.06)

## Discussion

Sow milk yield is a major determinant for the growth rate of suckling piglets. Backfat thickness at farrowing, as a proxy for body reserves, has been associated with mammary gland development, milk production and piglet growth rate, especially in primiparous sows (Amdi et al., 2013; Farmer et al., 2017). With current genetic types, there is evidence of a positive relationship between sow backfat thickness and offspring growth (Amdi et al., 2014; Rempel et al., 2015; Farmer et al., 2017), but sows with too much backfat thickness in late gestation (>26 mm) may produce piglets with reduced growth rate (Farmer et al., 2017). In our experiment, sow backfat thickness at parity was around this upper limit, so we would expect either no effect or a negative effect of increased fatness on piglet performance, especially because TT sows did not show greater backfat or loin thickness loss throughout lactation. Here, we detect further evidence of the negative effect of TT sows on litter performance, in line with the results by Solé et al. (2021a), who, with a larger number of sows, found that piglets weaned from TT sows weighed 126 g less than those weaned from C − sows. However, in this latter study the maternal effect could be underestimated because the piglet genotype was unknown and TT piglets, which are expected to grow more rapidly (Solé et al., 2021b), are overrepresented in litters from TT sows. Considering the piglet genotype and only the non-adopted piglets, we confirmed both effects, i.e., that TT individuals exhibit a lower maternal capacity, but, in contrast, they benefit from a higher growth potential. This behavior can be explained because TT individuals are more efficient in accumulating body reserves than in releasing them when needed (Solé et al., 2021b). In terms of maternal abilities, this means that lactating TT sows have less recourse to body reserves to support pregnancy and milk production. The available data (70 piglets lost during lactation, mostly crushed, and low viable) were insufficient to establish a conclusive relationship between genotype and mortality, but, in line with Solé et al. (2021a), results here did not evidence a strong effect of the sow genotype on preweaning mortality (9.4%, for TT sows, and 9.3%, for C − sows). Unfortunately, we did not have access to genotype lost piglets, so we could not explore the potential influence of the piglet genotype on pre-weaning mortality.

The beneficial effect of the TT genotype on growth was already detected at birth, although it was only overtly evidenced at weaning. Conversely, piglet sex affected BW but not WW, in agreement with previous findings that males are heavier than females at birth but not at weaning (Škorjanc et al., 2007; Baxter et al., 2012; Zindove et al., 2021). In particular, Zindove et al. (2021) reported that female piglets are more likely to be the heaviest and lightest within their litters at weaning. However, it should be noted that here males were castrated at 5 to 7 d of age, which could have affected their suckling and growing capacity (McGlone et al., 1993). In spite of this, field results indicate that castrated males at this age are weaned at an average weight similar to intact males (Morales et al., 2017). Thus, the effect of the *LEPR* genotype increased from around half the effect of sex, at birth, to more than 3-fold higher, at weaning. Manipulation of the sex ratio (proportion of males) by sows can be viewed as an adaptative way to optimize reproductive returns. Here, sows had more males than females at birth (54.1%, for TT sows, and 53.3%, for C − sows) and, to lesser extent, at weaning (50.8% of non-adopted piglets, for TT sows, and 52.7%, for C − sows). These results are consistent with those in Baxter et al. (2012), who suggested that sows provide more males at birth in anticipation of greater pre-weaning male losses. Since increased mortality in males is associated with higher energy demands, it can be hypothesized that male-bias at birth could be higher in TT sows in anticipation of their lower mothering ability. In addition to differences in sow resource allocation, a piglet’s body weight depends on its genetically drive for growth. Sferruzzi et al. (2016) showed that the placenta is able to fine-tune the supply of maternal resources to the fetus in accordance with the fetal genetic background. The TT piglets could take more advantage of this placenta ductility given that placental leptin is an important fetal growth factor (Ashworth et al., 2000).

Unlike what happens with males, TT piglets are able to sustain the difference in BW up to weaning. Besides higher BW, there are other reasons why TT piglets could perform better in lactation. Homeothermy and immune-competence are two of them. According to Baxter et al. (2012), males fail to maintain their growth advantage at weaning because they would have impaired thermoregulatory abilities as compared to females and thus chilling will predispose them more to starvation and disease. Reasoning in reverse, we can hypothesize that TT piglets may have enhanced homeothermy and immune-competence through increased feed intake and fat accumulation (Solé et al., 2021b). The immune system of the newborn piglet is immature and consequently the procurement of colostrum after birth is essential. In addition to BW, neonatal vitality can be used as a proxy to the capacity of a piglet to reach the udder and feed. Our results indicate that TT piglets are more vital at birth and, as piglet females, they have a more active suckling behavior. Baxter et al. (2012) reported that females were more vigorous than males at birth, suggesting that this attitude impel them to perform adequate suckling and thus compensate in lactation the weight advantage of males at birth. However, we do not find a relevant relationship of VIT with BW and WW, likely because VIT scores based on behavioral observations are more influential on the survival of susceptible piglets than on piglet growth (Baxter et al., 2008, 2012; Muns et al., 2013). For the purpose of this study, we only considered non-adopted piglets, which were on average 230 g heavier at birth than adopted piglets. This may have altered the relationship between VIT and WW and biased downwards the difference between TT and C − piglets for WW. Weighting less at birth, C − piglets were adopted more frequently (9.8% vs 7.0%) and therefore a higher proportion of low-weight C − piglets was discarded for the analyses.

Although the vitality test used here was not useful in predicting piglet performance during lactation, it was capable of capturing behavioral differences between *LEPR* genotypes. In particular, the outcome of the test indicates that TT piglets are more vital at birth. The defective T allele causes piglets to feel less readily satiated because, to reach that point, they require more circulating leptin (Ros-Freixedes et al., 2016) to counteract the leptin resistance-like state triggered by the leptin receptor deficiency (Lee et al., 1996). The C > T substitution takes place at exon 15 in the three transcripts currently annotated for the pig *LEPR* gene, resulting in a Leu to Phe amino acid substitution. In fact, functional evidence indicates that the T allele reduces leptin signaling through decreased transcript abundance and stability (Óvilo et al., 2010; Pérez-Montarelo et al., 2013). Therefore, a plausible interpretation of the higher vitality of TT piglets is that appetite rather than vigorousness per se prompts them to be more competitive and thus move rapidly to reach the udder, which is consistent with the fact that, if unsatisfied (i.e., not inactive), TT piglets, as compared to C − littermates, are more likely to be suckling than wandering. Newborn piglets compete for access to the teats and the most intense fights usually happen at the very beginning of lactation (de Passille and Rushen, 1989), when colostrum is produced. Competition for colostrum could be of vital importance (Quesnel et al., 2012), so stronger competitors (i.e., heavier and more vital) have a better chance of getting early immunoglobin-rich colostrum (Skok and Škorjanc, 2014). In fact, the number of fights for a teat that a piglet wins on the first day of lactation has been positively related to subsequent weight gain (de Pasillé et al., 1988). In conclusion, our findings provide evidence that the effects of *LEPR*, and by extension probably also those of other appetite-influencing genes, are already observable at birth and have both developmental and behavioral implications.

## Abbreviations:

BW: birth weight
LEPR: leptin receptor
ST: status of the piglet before vitality test
WG: weight gain during lactation
VIT: vitality
WW: weaning weight
